# Complexity for Artificial Substrates (*CASU*): Software for Creating and Visualising Habitat Complexity

**DOI:** 10.1371/journal.pone.0087990

**Published:** 2014-02-14

**Authors:** Lynette H. L. Loke, Nicholas R. Jachowski, Tjeerd J. Bouma, Richard J. Ladle, Peter A. Todd

**Affiliations:** 1 Experimental Marine Ecology Laboratory, Department of Biological Sciences, National University of Singapore, Singapore, Singapore; 2 Department of Geography, National University of Singapore, Singapore, Singapore; 3 Marine and Coastal Systems, Deltares, Delft, The Netherlands; 4 Royal Netherlands Institute for Sea Research - NIOZ Yerseke, Yerseke, The Netherlands; 5 Institute of Biological and Health Sciences, Federal University of Alagoas, Maceio, Brazil; 6 School of Geography and the Environment, Oxford University, Oxford, United Kingdom; University of Sydney, Australia

## Abstract

Physical habitat complexity regulates the structure and function of biological communities, although the mechanisms underlying this relationship remain unclear. Urbanisation, pollution, unsustainable resource exploitation and climate change have resulted in the widespread simplification (and loss) of habitats worldwide. One way to restore physical complexity to anthropogenically simplified habitats is through the use of artificial substrates, which also offer excellent opportunities to explore the effects of different components (variables) of complexity on biodiversity and community structure that would be difficult to separate in natural systems. Here, we describe a software program (*CASU*) that enables users to visualise static, physical complexity. *CASU* also provides output files that can be used to create artificial substrates for experimental and/or restoration studies. It has two different operational modes: simple and advanced. In simple mode, users can adjust the five main variables of informational complexity (i.e. the number of object types, relative abundance of object types, density of objects, variability and range in the objects’ dimensions, and their spatial arrangement) and visualise the changes as they do so. The advanced mode allows users to design artificial substrates by fine-tuning the complexity variables as well as alter object-specific parameters. We illustrate how *CASU* can be used to create tiles of different designs for application in a marine environment. Such an ability to systematically influence physical complexity could greatly facilitate ecological restoration by allowing conservationists to rebuild complexity in degraded and simplified habitats.

## Introduction

Rapid urbanisation, resource exploitation and climate change have resulted in the loss of habitats and species across ecosystems worldwide. As potential impacts become reality, three broad and interlinked fields of study have arisen in response: ‘reservation’, ‘restoration’, and ‘reconciliation’ ecology. All have the general aim of ameliorating the negative effects of human activity on the natural world, but their foci are different: to maintain and preserve biodiversity if not already lost (reservation), increase/restore/rehabilitate structure and function if degraded (restoration), or to enhance/input biodiversity in human-modified habitats if it is low or absent (reconciliation). Common to all three, although not often emphasized, is the problem of increasing simplification of ecosystems across all spatiotemporal scales. Even though structural simplification can take place naturally, anthropogenic simplification is far more frequent and rapid. Indeed, simplification of natural habitats (e.g. transformation of native forests into monocultures or replacement of natural shorelines with artificial seawalls) and their subsequent restoration is a major conservation challenge [1]. This is because physical habitat complexity regulates the structure and function of biological communities, although the mechanisms involved remain unclear [2], [3]. Research in this critical area of study, however, is hindered by the ambiguity regarding the definition of ‘complexity’ [4]–[6]. This lack of clarity and precision has significantly handicapped efforts to measure or artificially create complexity, and has even influenced how conclusions from ‘complexity studies’ are drawn and interpreted.

There is a growing consensus that influencing complexity is likely to be critical for restoration efforts (e.g. [7], [8]), partly because it is far more tractable to manipulation than many of the other factors known to affect biodiversity [9]. One way to increase complexity during ecological restoration is through the use of artificial substrates [10]. For example, a wide spectrum of man-made substrates across a range of sizes, from small settlement tiles or cement plugs to large modular structures have been utilised in marine restoration work (e.g. [11]–[13]). Many of these substrates aim to augment biodiversity through the incorporation of some form of ‘topographic complexity’; but this is challenging because the majority of metrics currently available are more suitable for quantifying complexity rather than guiding the (re)creation of complex habitats. While common metrics such as fractal dimensions may be useful for measuring complexity in the field, it is impracticable to translate or convert these numbers into ecologically relevant and practical solutions for restoration.

The problem of defining complexity has led to widespread confusion and conceptual stagnation concerning its role/mechanism(s) in biological communities [14]. Notwithstanding the lack of a definition, it is also difficult to empirically separate the different aspects of complexity (e.g. increasing surface area with increasing complexity) [15]. Most ‘complexity studies’ can be split into two essential forms: *systems-based* and *information-based* (or informational) complexity. Systems-based complexity can be defined as the unexpected and/or unpredictable emergent properties that arise from the interactions between much simpler components, such that the overall properties of the complex system are not obvious from the properties of the individual components–this usually involves a temporal element. Informational complexity, on the other hand, is based on information theory and has no temporal component. The fundamental premise is that the greater the informational content, the greater the complexity [16]. The ‘subject’ can be anything of interest, living or non-living. Knowingly or not, most habitat ‘complexity studies’ examine the informational content of the study system (components of a system at fixed time points), with complex habitats containing more ‘information’ than simple ones. The most commonly used measure of informational complexity is Shannon’s entropy which calculates information content, that is, the entropy or degree of uncertainty associated with a random variable [16].

Complexity-diversity relationships are often examined by measuring the amount of information content of a subject of interest, but no studies and/or software to date are available for converting these metrics into viable and rigorous solutions for restoration and reconciliation work. Hence, we devised a programme ‘*CASU*’ that can be used to both visualise ‘informational complexity’ and design artificial substrates with varying (controllable) levels of complexity (please refer to Appendix S1 & S2 for programme and user manual).

### Overview of *CASU:* Software for Creating and Visualising Habitat Complexity

**Figure 1:**
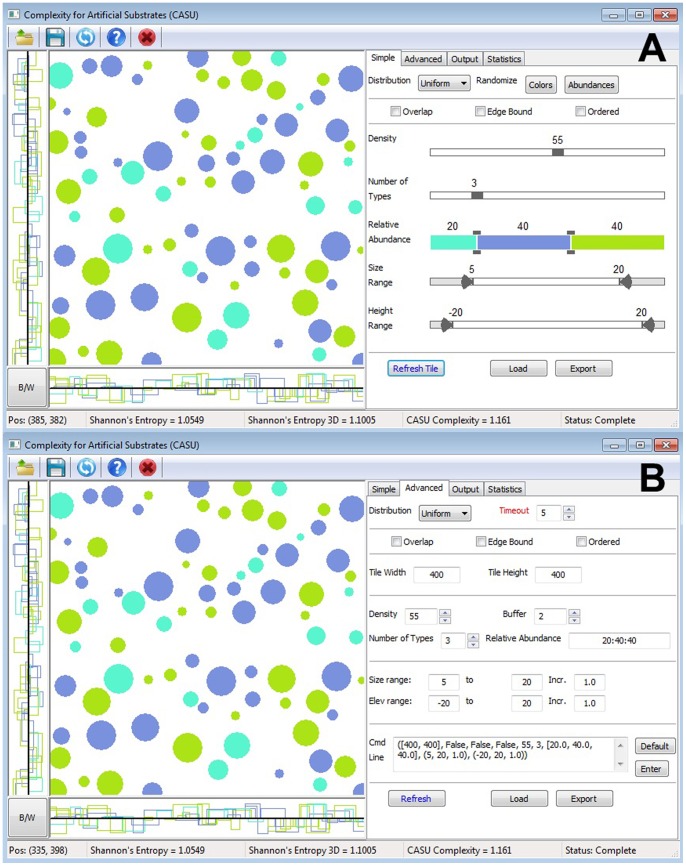
Screenshot of *CASU*. *CASU* in (A) ‘simple mode’ and (B) ‘advanced mode’.

*CASU* was originally conceived and developed as part of a project to increase biodiversity on seawalls using artificial substrates, i.e. moulded concrete tiles. In particular, we wanted to compare colonisation of ‘simple’ and ‘complex’ tiles of the same surface area. As concrete tiles are patently not dynamic, only informational complexity could be incorporated into their topography. Shannon’s entropy was adopted for creating informational complexity as it quantifies the uncertainty in predicting the object type of a component that is taken at random from the set. For a random variable X with the distribution (*p*_1_, …, *p*_n_), the Shannon’s entropy of the random variable denoted by H(X) is therefore defined as:
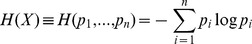
where *p*_i_ is the probability mass function of *i*th outcome [17], [18]. Thus, the greater the number of object types, and the more equal their proportional abundances are, the more difficult it is to correctly predict which component will be present on any particular part of the tile surface. Also, there are a finite number of ways the topography of a tile can be altered, these are: (#1) the number of object types (within *CASU* each ‘object’ is represented by a circle and different colours represent different ‘object types’–for more details, please see user manual; Appendix S2), (#2) the relative abundance of each object type, (#3) the density of objects, (#4) the variability and range in the objects’ dimensions (e.g., length, width and height), and (#5) spatial arrangement of the objects. These five variables comprise the main features of *CASU* when operating in its ‘simple’ mode and users can make changes to any of them (Figure 1A). Software settings are reflected on the tile surface, facilitating easy visualisation of the concepts and components of informational complexity (Figure 1).

The ratio for each object type (relative abundance, as represented by different colours), may be randomised with each tile generation. Informational complexity increases directly with a greater number of objects types (#1), evenness (as adjusted via the relative abundance setting) (#2) and density (#3). Increasing the size range of object types (#4) however, has no effect on informational complexity when components are chosen from a continuous probability distribution. This makes comparisons between tiles inappropriate, as the probability of selecting any size value from such a set is infinite. However, comparisons of complexity are workable when selecting from a discrete probability distribution; tiles whose size ranges have smaller increment values have greater complexity than tiles with large increment values (within the same size range).

What is often called ‘heterogeneity’ or ‘spatial heterogeneity’ in ecological literature refers to the number of different object types (#1) and their variability (#4). Even though greater density (#3) adds directly to the informational content, the effect of heterogeneity can dominate the effect of density by virtue of it being a higher order factor. For instance, a tile with 2 components ( = density) and 5 descriptors ( = heterogeneity) will have a total of 32 possible combinations (i.e. 2 components to the power of 5 descriptors) but a tile with 5 components and 2 descriptors will only have 25 possible combinations (i.e. 5 components to the power of 2 descriptors).

The addition of rules (such as the spatial arrangement of objects on the tiles; #5) also has an effect on informational complexity. In informational complexity, rules reduce the amount of information required to encode the data; thus a randomised arrangement will be more complex than one following some rules or pattern (e.g. ordered). *CASU* does not take into account, or offer control of, possible interactions among component types (such as clumping) as it treats each component as non-living objects. *CASU* is also scale-free, which permits users to extrapolate the generated output to their preferred or relevant scale. Although Shannon’s entropy is calculated based on the numerical parameters, caution must be exercised when comparing these values (i.e. using it as a proxy for informational complexity) as they are only meaningful when comparing tiles with the same rules (for instance, between ordered and random tiles, or between tiles whose component sizes were chosen from either infinite continuous or finite discrete sets).

As we designed *CASU* for building complexity into artificial substrates, an advance mode was included in the programme where users can manually change the parameters that were displayed in the simple mode, including a buffer (i.e., minimum spacing) between each object on the tile (Figure 1B). In the output, they may also change the position and size of a specific object or delete it entirely. Finally, output files are optimised for Microsoft Excel and computer-aided design (CAD) software, (e.g. AutoCAD) so that designs can be used to create actual substrates (please see Appendix S2).

### Examples of How *CASU* can be Applied

Lundholm and Richardson [19∶966] highlighted that “abiotic and biotic differences between artificial analogues and natural systems can be frequently overcome by ecological engineering to make the environment more suitable for native biodiversity”. This statement underlines the huge potential for reconciliation ecology, i.e. the modification of anthropogenic habitats to give some species back their geographic ranges while humans still retain theirs [20]. However, scientists and managers engaged in restoration and reconciliation work often do not have the tools for designing or re-designing novel habitats to enhance habitat complexity and this is reflected in the trial-and-error approach that many studies adopt. Below, we illustrate some of the potential applications of *CASU* in coastal environments.

#### Manipulating topographic complexity for enhancing biodiversity on seawalls

Worldwide, coastal areas are increasingly becoming urbanised [21], [22], resulting in the extensive alteration of natural shorelines with jetties, pier-pilings, pontoons and breakwaters; or their wholesale replacement with seawalls and similar defences [23]. Despite the proliferation of foreshore artificial structures, relatively few studies have examined the biological communities inhabiting these novel environments or the ecological impacts of such coastal modifications [24]. Among all urban coastal structures seawalls are the most extensive, but they tend to support less diverse intertidal communities relative to natural shores [25]–[29]. They are characterised by the reduction of various microhabitats (e.g. pits, rock-pools, crevices and overhangs) and low topographic complexity, both of which are usually negatively correlated with taxa richness [22], [30], [31].

**Figure 2:**
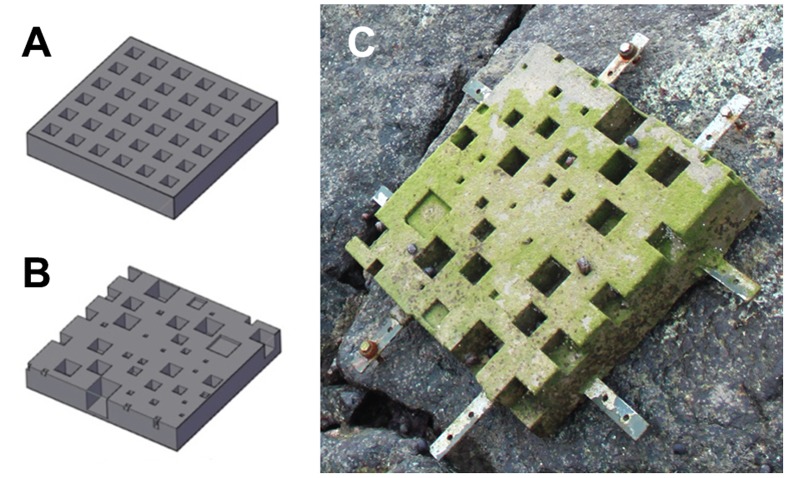
3D models (AutoCAD drawings) of tiles with a single structural component (square-pits) at two levels of complexity generated via *CASU*. (A) ‘simple tile’ and (B) ‘complex tile’. (C) a fabricated 40×40×6 cm^3^ concrete tile mounted onto a seawall (photograph taken one month after deployment).

In early 2011 a project was initiated to examine how seawalls around Singapore may be engineered using artificial substrates to enhance their biodiversity. Since relevant living spaces (or microhabitats) are often a limiting factor for species diversity, especially at small spatial scales, restoration and reconciliation methods can exploit the role of habitat complexity to achieve the goal of increasing biodiversity [1], [8], [32]. Using a pilot version of *CASU*, it was possible to test whether topographically more complex substrates can support greater diversity by designing two types of concrete tiles (40×40×6 cm^3^) one structurally more complex than the other, but with equal surface areas (Figure 2). To create a pitted ‘simple tile’, the width, length, depth and spacing of all pits was fixed and arranged in an ordered formation on a tile using *CASU*. The fixed value was then used as the mean (of a range of values following a discrete probability distribution) when randomly varying the size, depth and spacing of each component for the ‘complex tile’. Granite control tiles were also constructed to mimic the surface of a seawall. Unpolished slabs of granite were broken up and cemented onto a concrete base to recreate the cracks and crevices found on seawalls around Singapore.

Five replicates of each tile type were attached randomly onto granite seawalls (at a low shore height) at two islands south of Singapore Island (Figure 2C), creating a two-way ANOVA design with ‘Site’ and ‘Tile type’ as factors. After 13 months of colonization, all the tiles were collected and their assemblages compared. Our preliminary results suggest that greater structural complexity (at the 8–56 mm scale tested) can support higher diversity that is independent of surface area.

## Discussion

Many studies describe a positive relationship between habitat complexity and biodiversity (e.g. [33], [34]); possibly due to a greater number of niches and/or resource partitioning (e.g. [35], [36]). This has been noted in both terrestrial [35]–[37] and aquatic systems (e.g. [38], [39]), but complexity is measured in very different ways between and within these environments (see review of techniques by [14]). Even though complexity has been closely tied to community persistence and ecosystem stability and functioning [3], a mechanistic understanding of its role in structuring communities is lacking, with relatively few studies examining the effects of complexity on community and ecosystem properties. Furthermore, the imprecise use of terms such as ‘complexity’ and ‘heterogeneity’ has hindered our understanding of how the number of species is related to, and regulated by, complexity–yet this knowledge is essential for mitigating the effects of habitat modification or loss [40], [41].

*CASU* was developed in an effort to visualise and create complexity. The potential advantages of adopting this approach are considerable: (i) habitat complexity can be precisely manipulated within experimental settings, allowing the influence of complexity on species richness to be carefully controlled and compared; (ii) complexity can be augmented to different degrees in order to increase species richness within restoration projects. Even though such augmentation has been attempted (e.g. [1], [32]) it has so far taken place arbitrarily, for example, by adding substrate such as stones and boulders to create new microhabitats in streambeds, or reconstructing and restoring channel complexity by re-meandering rivers and streams [1], [32]. Software applications such as *CASU* have the potential to standardise the manipulation of complexity within restoration initiatives, enhancing comparability and allowing more powerful statistical evaluations; (iii) by adopting a standardised metric of information complexity (such as Shannon’s entropy) to measure, compare and create habitat complexity, researchers from different sub-fields can share applications (and a common language), greatly promoting the rate of progress in understanding the role of habitat complexity in structuring ecosystem processes and ecological assemblages. It is important to note, however, that no compound measure can encapsulate all aspects of complexity, and hence additional information (in this case, *CASU* output such as the number of object types, relative abundance of object types, density of objects, variability and range in the objects’ dimensions, and their spatial arrangement, e.g. random vs ordered) is required for robust comparisons. Furthermore, experimenters should be explicit regarding the models and hypotheses that they are testing.

Experiments and restoration projects often utilise concrete for fabricating artificial substrates because of its availability, versatility, low cost and ease of use. Concrete is one of the few viable ways of creating these substrates on a large scale, allowing researchers and engineers to progress from the small-scale efforts characteristic of academic research to the large-scale needs of practical restoration/reconciliation efforts. *CASU* was developed to design complex moulds for concrete at any scale desired. Designs can be adapted for restoration, reconciliation, as well as empirical research on the effects of physical complexity. Our examples involving ‘simple’ and ‘complex’ concrete tiles illustrate how this can be achieved. However, the application of *CASU* does not have to be limited to moulded concrete substrates. Spatial randomisation of components and object types can be used in other scenarios, such as tree planting (where tree species are the object types) or the size and arrangement of artificial pools to enhance amphibian populations. As “differential habitat selection is one of the principal relationships which permit species to co-exist” [42∶327], we expect that most reconciliation efforts will require a means of incorporating some aspect of physical habitat complexity into anthropogenic habitats–which tend to be structurally quite simple.

*CASU* also serves as a visualisation tool for informational complexity. By having all the variables of complexity represented on a tile surface, it is easy to see how each variable contributes to the overall complexity of the tile (Figure 1). This may be helpful for distinguishing the different aspects of complexity within a research design, as the term is used variably in the current literature. While it is desirable to have a single metric to encapsulate the multidimensionality of informational complexity, it is not feasible using the approach we have adopted. The problem is very similar to reporting Shannon-Wiener diversity index (*H’*); the number alone has limited use as it can be achieved in different ways. For each site, *H’* needs to be accompanied by information on species richness, total abundance, and some indication of evenness to give a more complete picture. Thus, although we included the entropy value (calculated from the numerical input only) in *CASU*, users should not assume that this provides a definitive measure of complexity. Like *H’*, it needs to be accompanied by a description of the other aspects of complexity, especially the number of component types and their density.

Frequent calls are made for more research on the role of complexity in ecology; for instance, in the design of reserves [43], [44], the preservation of ecosystem functions [45], and maintenance of threatened species [46]. As urbanisation spreads across the globe [47], restoration and reconciliation ecology is likely to play an increasingly important role in maintaining biodiversity. Retrofitting artificial substrates, or incorporating biodiversity-enhancing designs into new projects, are two potential strategies that are recognised by conservation biologists, but which are not yet fully developed or utilised. *CASU* contributes by demystifying complexity, while providing a tool for creating it.

## Supporting Information

Appendix S1 **Complexity for artificial substrates (** ***CASU*** **) programme.**
(ZIP)

Appendix S2 ***CASU*** **: User manual.**
(DOC)

## References

[1] Larkin D, Vivian-Smith G, Zedler JB (2006) Topographical heterogeneity theory and ecological restoration. In: Falk DA, Palmer MA, Zedler JB, editors. Foundations of restoration ecology. Washington, DC: Island Press. 142–164.

[2] Pianka ER (2000) Evolutionary ecology, 6^th^ edition. San Francisco: Benjamin Cummings.

[3] Cardinale BJ, Palmer MA, Collins SL (2002) Species diversity enhances ecosystem functioning through interspecific facilitation. Nature 415: 426–429. 11807553 10.1038/415426a

[4] Kovalenko KE, Thomaz SM, Warfe DM (2012) Habitat complexity: approaches and future directions. Hydrobiologia 685: 1–17.

[5] Loehle C (2004) Challenges of ecological complexity. Ecol Complex 1: 3–6.

[6] Tews J, Brose U, Grimm V, Tielbörger K, Wichmann MC, et al (2004) Animal species diversity driven by habitat heterogeneity/diversity: the importance of keystone structures. J Biogeogr 31: 79–92.

[7] Palmer MA, Menninger HL, Bernhardt E (2009) River restoration, habitat heterogeneity and biodiversity: a failure of theory or practice?. Freshwater Biol 55: 1–18.

[8] Kovalenko KE, Dibble ED, Slade JG (2010) Community effects of invasive macrophyte control: role of invasive plant abundance and habitat complexity. J Appl Ecol 47: 318–328.

[9] Bell SS, Fonseca MS, Motten LB (1997) Linking ecological restoration and landscape ecology. Restor Ecol 5: 318–323.

[10] Matias MG, Underwood AJ, Hochuli DF, Coleman RA (2010) Independent effects of patch size and structural complexity on diversity of benthic macroinvertebrates. Ecology 91: 1908–1915. 20715609 10.1890/09-1083.1

[11] Pickering H, Whitmarsh D (1997) Artificial reefs and fisheries exploitation: a review of the ‘attraction versus production’ debate, the influence of design and its significance for policy. Fish Res 31: 39–59.

[12] Burt J, Bartholomew A, Usseglio P, Bauman A, Sale PF (2009) Are artificial reefs surrogates of natural habitats for corals and fish in Dubai, United Arab Emirates? Coral Reefs 28: 663–675.

[13] Guest JR, Heyward A, Omori M, Iwao K, Morse A, et al. (2010) Rearing coral larvae for reef rehabilitation. In: Edwards AJ, editor. Reef Rehabilitation Manual. Saint Lucia: Coral Reef Targeted Research & Capacity Building for Management Program. 73–92.

[14] Kovalenko KE, Thomaz SM, Warfe DM (2012) Habitat complexity: approaches and future directions. Hydrobiologia 685: 1–17.

[15] Johnson MP, Frost NJ, Mosley MWJ, Roberts MF, Hawkins SJ (2003) The area-independent effects of habitat complexity on biodiversity vary between regions. Ecol Lett 6: 126–132.

[16] Mitchell M (2009) Complexity: a guided tour. New York: Oxford University Press.

[17] Shannon CE (1948) A mathematical theory of communication. Bell Syst Tech J 27: 379–423, 623–656.

[18] Ihara S (1993) Information theory for continuous systems. London: World Scientific Publishing.

[19] Lundholm JT, Richardson PJ (2010) Habitat analogues for reconciliation ecology in urban and industrial environments. J Appl Ecol 47: 966–975.

[20] Rosenzweig ML (2003) Reconciliation ecology and the future of species diversity. Oryx 37: 194–205.

[21] Moschella PS, Abbiati M, Åberg P, Airoldi L, Anderson JM, et al (2005) Low-crested coastal defence structures as artificial habitats for marine life: Using ecological criteria in design. Coast Eng 52: 1053–1071.

[22] Chapman MG, Blockley DJ (2009) Engineering novel habitats on urban infrastructure to increase intertidal biodiversity. Oecologia 161: 625–635. 19551409 10.1007/s00442-009-1393-y

[23] Bulleri F, Chapman MG, Underwood AJ (2004) Patterns of movement of the limpet *Cellana tramoserica* on rocky shores and retaining seawalls. Mar Ecol Prog Ser 281: 121–129.

[24] Chapman MG, Bulleri F (2003) Intertidal seawalls–new features of landscape in intertidal environments. Landsc Urban Plan 62: 159–172.

[25] Chapman MG (2006) Intertidal seawalls as habitats for molluscs. J Molluscan Stud 72: 247–257.

[26] Browne MA, Chapman MG (2011) Ecologically informed engineering reduces loss of intertidal biodiversity on artificial shorelines. Environ Sci Technol 45: 8204–8207. 21875080 10.1021/es201924b

[27] Firth LB, Thompson RC, White FJ, Schofield M, Skov MW, et al (2013) The importance of water-retaining features for biodiversity on artificial intertidal coastal defence structures. Divers Distrib 19: 1275–1283.

[28] Pister B (2009) Urban marine ecology in southern California: the ability of riprap structures to serve as rocky intertidal habitat. Mar Biol 156: 861–873.

[29] Gacia E, Satta MP, Martin D (2007) Low crested coastal defence structures on the Catalan coast of the Mediterranean Sea: how they compare with natural rocky shores. Sci Mar 71: 259–267.

[30] Borsje BW, van Wesenbeeck BK, Dekker F, Paalvast P, Bouma TJ, et al (2011) How ecological engineering can serve in coastal protection. Ecol Eng 37: 113–122.

[31] Bulleri F, Chapman MG (2004) Intertidal assemblages on artificial and natural habitats in marinas on the north-west coast of Italy. Mar Biol 145: 381–391.

[32] Spänhoff B, Arle J (2007) Setting attainable goals of stream habitat restoration from a macroinvertebrate view. Restor Ecol 15: 317–320.

[33] Clough Y, Barkmann J, Juhrbandt J, Kessler M, Wanger TC, et al (2011) Combining high biodiversity with high yields in tropical agroforests. P Natl Acad Sci USA 108: 8311–8316. 10.1073/pnas.1016799108 PMC3100989 21536873

[34] Huston M (1979) A general hypothesis of species diversity. Am Nat 113: 81–101.

[35] Pianka ER (1966) Convexity, desert lizards, and spatial heterogeneity. Ecology 47: 1055–1059.

[36] Holzschuh A, Steffan-Dewenter I, Klein D, Tscharntkei T (2007) Diversity of flower-visiting bees in cereal fields: effects of farming system, landscape composition and regional context. J Appl Ecol 44: 41–49.

[37] Kerr JT, Packer L (1997) Habitat heterogeneity as a determinant of mammal species richness in high-energy regions. Nature 385: 252–254.

[38] Hovel KA, Lipcius RN (2001) Habitat fragmentation in a seagrass landscape: patch size and complexity control blue crab survival. Ecology 82: 1814–1829.

[39] Moore EC, Hovel KA (2010) Relative influence of habitat complexity and proximity to patch edges on seagrass epifaunal communities. Oikos 119: 1299–1311.

[40] Bulleri F (2005) Experimental evaluation of early patterns of colonisation of space on rocky shores and seawalls. Mar Environ Res 60: 355–374. 15769504 10.1016/j.marenvres.2004.12.002

[41] Chapman MG, Underwood AJ (2011) Evaluation of ecological engineering of “armoured” shorelines to improve their value as habitat. J Exp Mar Biol Ecol 400: 302–313.

[42] Rosenzweig ML (1981) A theory of habitat selection. Ecology 62: 327–335.

[43] Dobkin DS, Olivieri I, Ehrlich PR (1987) Rainfall and the interaction of microclimate with larval resources in the population dynamics of checkerspot butterflies (*Euphydryas editha*) inhabiting serpentine grassland. Oecologia 71: 161–166. 28312241 10.1007/BF00377280

[44] Miller RI, Bratton SP, White PS (1987) A regional strategy for reserve design and placement based on an analysis of rare and endangered species’ distribution patterns. Biol Conserv 39: 255–268.

[45] Ludwig JA, Tongway DJ (1996) Rehabilitation of semiarid landscapes in Australia. II: Restoring vegetation patches. Restor Ecol 4: 398–406.

[46] Fleishman E, Launer AE, Weiss SB, Reed JM, Boggs CL, et al (2000) Effects of microclimate and oviposition timing on prediapause larval survival of the Bay checkerspot butterfly, *Euphydryas editha bayensis* (Lepidoptera: Nymphalidae). J Res Lepidoptera 36: 31–44.

[47] May RM (2007) Unanswered questions and why they matter. In: May RM, McLean AR, editors. Theoretical Ecology: principles and applications, 3^rd^ edition. New York: Oxford University Press. Pp. 205–215.

