# Supplementary figures and images for: Complexity for Artificial Substrates (CASU): Software for Creating and Visualising Habitat Complexity
Source: PLoS One. 2014 Feb 14;9(2):e87990. doi: 10.1371/journal.pone.0087990 (PMC3925107; doi:10.1371/journal.pone.0087990)

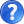

Supplement: Appendix S1 — Complexity for artificial substrates ( CASU ) programme. (ZIP) [file pone.0087990.s001.zip › CASU program/help.png]

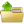

Supplement: Appendix S1 — Complexity for artificial substrates ( CASU ) programme. (ZIP) [file pone.0087990.s001.zip › CASU program/open.png]

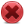

Supplement: Appendix S1 — Complexity for artificial substrates ( CASU ) programme. (ZIP) [file pone.0087990.s001.zip › CASU program/quit.png]

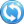

Supplement: Appendix S1 — Complexity for artificial substrates ( CASU ) programme. (ZIP) [file pone.0087990.s001.zip › CASU program/refresh.png]

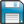

Supplement: Appendix S1 — Complexity for artificial substrates ( CASU ) programme. (ZIP) [file pone.0087990.s001.zip › CASU program/save.png]

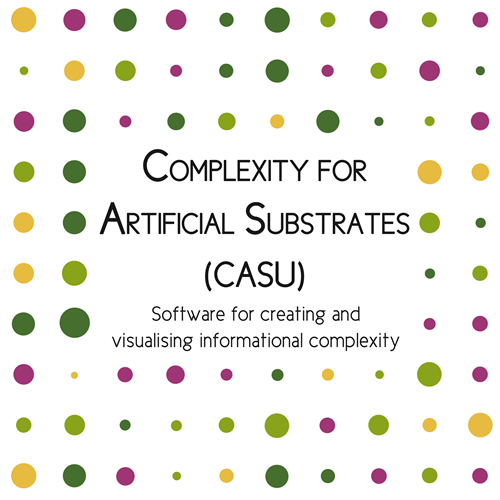

Supplement: Appendix S1 — Complexity for artificial substrates ( CASU ) programme. (ZIP) [file pone.0087990.s001.zip › CASU program/splash500.png]
