# Supplementary material for: Complexity for Artificial Substrates (CASU): Software for Creating and Visualising Habitat Complexity
Source: PLoS One. 2014 Feb 14;9(2):e87990. doi: 10.1371/journal.pone.0087990 (PMC3925107; doi:10.1371/journal.pone.0087990)
Supplement: Appendix S2 — CASU : User manual. (DOC) [file pone.0087990.s002.doc]

**Complexity for Artificial Substrates (*CASU*):** *software for creating and visualizing habitat complexity*

**User Manual**


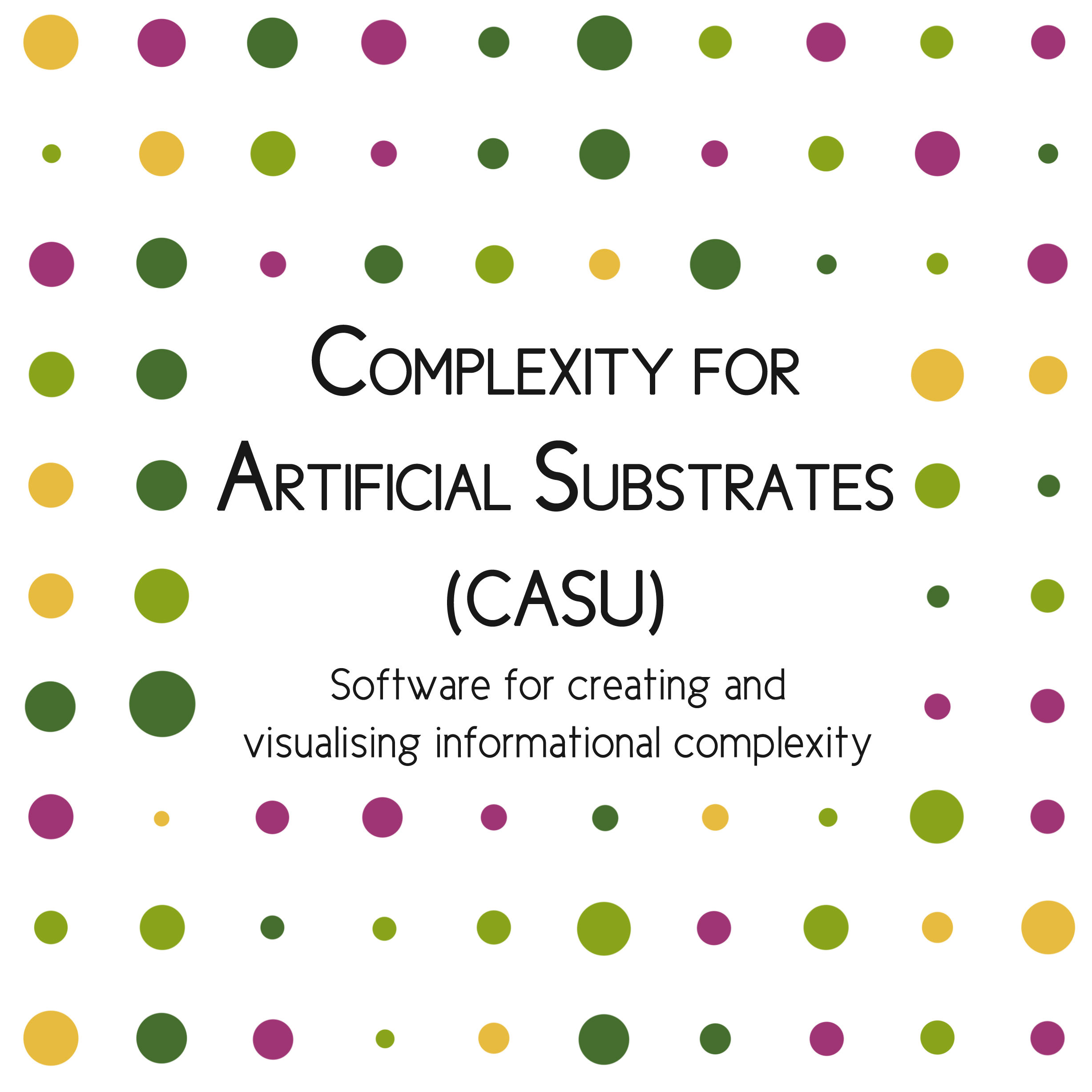


Lynette H. L. Loke, Nick R. Jachowski, Tjeerd J. Bouma,

Richard J. Ladle & Peter A. Todd

***CASU*: User Manual**

CONTENTS Page

1. Contact details and installation of *CASU* software 3

2. Introduction to *CASU* 4

3. Program Overview 5

4. Basic Functions 6

5. Substrate Viewer 8

6. Simple Menu 9

7. Advanced Menu 13

8. Output Menu 16

9. Statistics Menu 17

10. 3-Dimensional Modeling 18

11. Troubleshooting 19

12. Code Reference 19

13. Acknowledgements 20

14. References 20

**1. Contact details and installation of *CASU* software**

For any general enquires about *CASU* (or to report any bugs, technical problems, suggestions for improvement, etc.) please contact Peter A. Todd (*dbspat@nus.edu.sg*) or Lynette H. L. Loke (*lynetteloke@gmail.com*).

Institutional affiliation and present address:

Experimental Marine Ecology Laboratory,

Department of Biological Sciences,

National University of Singapore (NUS),

14 Science Drive 4, Blk S1 #02-02,

Singapore 117543

Tel: +65-65161034

Fax: +65-67792486

Computer Requirements: Currently only computers running Windows operating systems are supported. The program has been tested on Windows XP and Windows 7.

Download the Program: The program can be downloaded at:

Unzip the File: Newer versions of Windows (XP and newer) come pre-installed with unzipping software, otherwise please download a free program such as 7-zip to unzip the file. After being unzipped, the contents should be placed in a folder named ‘CASU’ if it is not already there by default. A common place to put this folder is in the ‘C’ directory or under ‘C:\Program Files\’, but it will run in any location.

Open the Program: Open the *CASU* folder and find the executable file called ‘CASU001.exe’. Double click this file to run the *CASU* program. Users preferring the executable file to be easily accessible from the Windows Start Menu should then right click on ‘CASU001.exe’ and select ‘Pin to Start Menu’.

**2. Introduction to *CASU***

Rationale: Positive correlations between species diversity and structurally complex habitats are frequently described in both terrestrial and marine systems, however, the mechanism by which complexity influences diversity remains unclear. Complex habitats potentially provide a wider range of available resources (e.g. microhabitats) which gives rise to a greater variety of ways in which they can be exploited, i.e. they offer a greater number of niches (Pianka, 2000; Ritchie & Olff, 2002). Simplification of natural habitats (e.g. transformation of native forests into monocultures) is a major conservation challenge and there is a growing consensus that influencing complexity is likely to be critical for restoration efforts (e.g. Palmer et al. 2009), partly because it is far more tractable to manipulation than many of the other factors known to affect biodiversity (Bell et al. 1997). One simple way to increase physical complexity during ecological restoration is through the use of artificial substrates (Matias et al., 2010), which have been successfully employed to study colonization and succession (e.g., see Cairns, 1982; Loke et al., 2010). Artificial substrates can also be used to mimic or augment certain important structural elements that have been removed from a habitat (Pratt 1994).

While there are various measures of complexity in ecology, there are presently no existing metrics that can be easily utilised to guide the design of artificial substrates with contrasting levels of informational complexity. Various quantitative measures of complexity exist (e.g. fractal dimensions) but they cannot be used to re-construct complexity on artificial substrates. Even though experimental studies involving artificial substrates often offer excellent opportunities to explore the effects of complexity variables that would be difficult to separate in natural systems without extensive manipulation, this is rarely performed in a rigorous way. Instead, substrates to enhance habitat complexity are often designed or chosen based on trial and error approaches that overlook key variables (e.g. spatial arrangement, component types or density) or fail to take into consideration scale-dependent aspects of the habitat to which the target community is responding. For example, area is rarely controlled for in studies involving more complex habitat treatments, thus observed increases in biodiversity may be confounded; the greater number of species observed in more complex habitats can often simply be due to the greater surface area sampled. *CASU* was designed primarily to allow users to create artificial substrates of different levels of complexity but similar surface area, which can be applied experimentally or used for restoration/reconciliation projects.

*CASU* produces a basic 2-D square tile (by default), which contains a number of circles (which we refer to as ‘Objects’). The different colours represent different ‘Object Types’; colours were used instead of patterns or shapes as they are neutral do not denote any meaning of complexity. For instance, if shapes were used to differentiate the types of objects, a triangle might be perceived as being more complex than a circle. However, users might adapt these ‘Object types’ to their own specific context (e.g. by substituting colours for different shapes). The main parameters of ‘informational complexity’ (i.e. density, number of object types, relative abundance, and size) which alter the complexity of the tile can be easily manipulated via a menu of sliders. There are also side-projections of the tile depicting a 3-D view of the depth and/or height of each object. *CASU* was originally conceived and developed to create small moulded concrete tiles but it can be used to help create designs at any scale.

3**. Program Overview**

The *CASU* program is composed of two main parts: the Substrate Viewer (on the left) and the Menu (on the right).

**
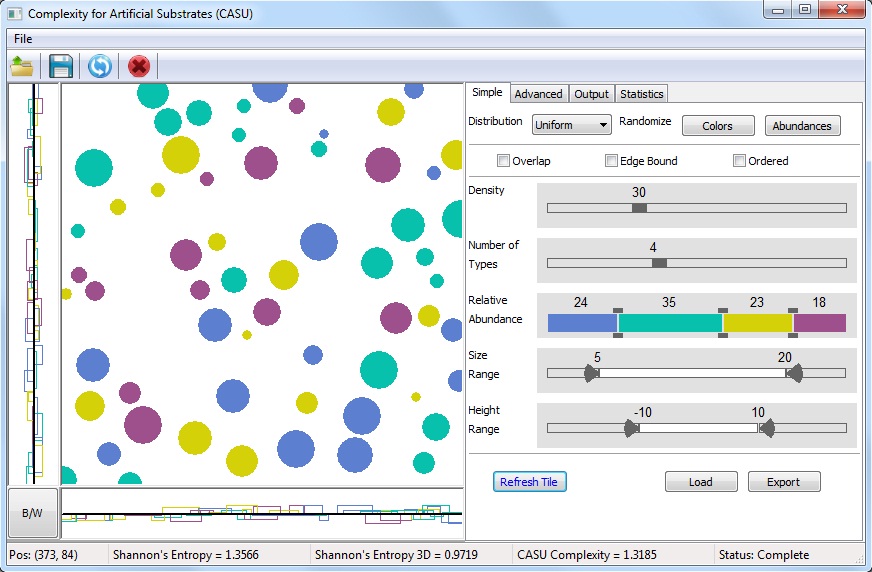
**

The Substrate Viewer shows the top and side views of the substrate created by the program. For more details, refer to the ‘Substrate viewer’ section on page 8.

The Menu is made up of 4 tabbed windows: Simple, Advanced, Output, and Statistics. These windows allow users to change the substrate parameters (Simple & Advanced) and to view information about the created substrate (Output & Statistics). For more details on each of these tabbed windows, refer to pages 9-17.

The program creates substrates based on the parameters and settings chosen from the menu. Every new substrate is created based on random variable distributions, therefore each substrate is unique. For more details about these distributions, refer to page 9.

Additionally, substrates can be manually tweaked to fine-tune particular properties.

The substrates can be saved and exported in various formats for later use in *CASU* or other imaging and analysis programs.

The program has an intuitive interface which we encourage users to explore. Adjusting the settings of the various buttons, sliders and entries is the quickest way to appreciate the variety of substrates that can be produced by *CASU*.

**4. Basic Functions**

Refresh: A new substrate can be created by clicking on the Refresh icon
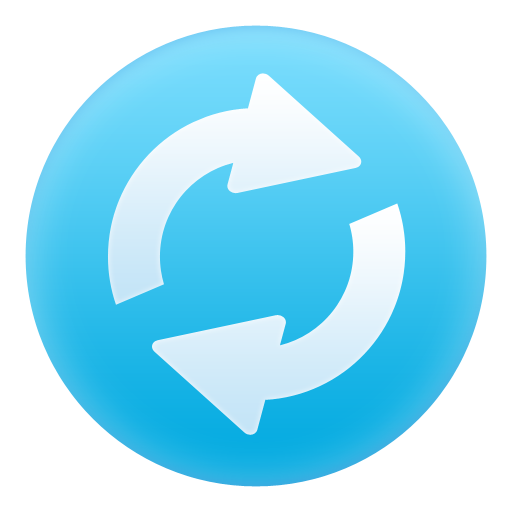
located above the substrate viewer or the Refresh Tile button at the bottom of the menu. Refresh creates a unique substrate every time by selecting new objects from the chosen parameter distributions, Substrates are not automatically saved before refreshing, so users should Save or Export the substrate if they wish to save it.

Save File: Files can be saved using the Save icon
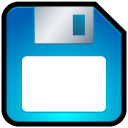
 located above the substrate viewer. The Save function produces four files. The filename given to the file at the time of saving is the same for all files, but the suffixes are different.

(1) filename.p - A python file containing the unique substrate details. This is the file which is used when a re-upload of the substrate to *CASU* is desired.

(2) filename.png - A top-view image of the substrate in PNG format. This can be opened and viewed using any image viewing software.

(3) filename.txt - A plain text file containing the substrate details such as the size and coordinates of each object. Additionally, it contains the parameters used to create the substrate. This file can be viewed using any text editor as well as imported into a spreadsheet program. If any changes are made to program default values these are reflected in the file header.

(4) filename.xls - A Microsoft Office Excel 97-2003 Worksheet containing the substrate details such as the size and coordinates of each object.

Export File: The Export button
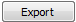
 at the bottom of the menu produces the same four files as the Save icon and an additional STL export file specifically for 3-D viewing and modeling. For more details on 3-Dimensional modeling, refer to page 18.

(5) filename.stl - The STL file can be used to view or create a 3-D model of the substrate. See the section on 3-D modeling for more information

Open File: Existing *CASU* files can be opened and loaded using the Open icon
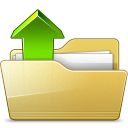
 located above the substrate viewer or the Load button
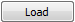
 at the bottom of the menu. Open and Load are identical in *CASU*. When the Open command is chosen, a dialog will appear prompting the selection of the file for *CASU* to load. This file must be of the type ‘filename.p’. Once the file is loaded the substrate window will display the substrate and the settings in the menu will reflect the substrate values and statistics.

Print File: The *CASU* software currently does not contain a function to print information, so any printing must be done using the exported files. These files can be opened and printed using common image viewer and text editing software.

Help: The Help button
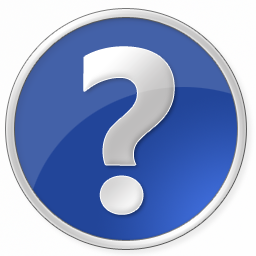
 links users directly to this manual.

Close: The *CASU* program can be exited using the Close icon
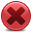
 located above the substrate viewer. Substrates are not automatically saved before exiting the program, so users should first Export the any substrate they wish to save.

**5. Substrate Viewer**

The Substrate Viewer is composed of four panels. The far left panel is a side view of the substrate. The main panel is a top view of the substrate. The bottom panel is another side view of the substrate. The bottom left panel is a button called ‘B/W’ which allows the user to toggle the substrate base color between black and white.

**
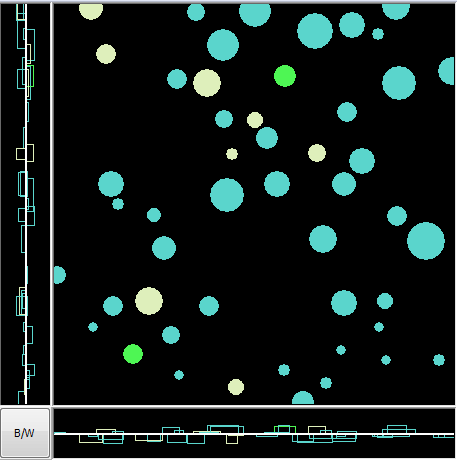
**

**6. Simple Menu**

The Simple Menu allows the user to set the basic parameters needed to make a substrate. It is designed to provide an instant visualisation of how complexity can be adjusted using these simple functions. The parameters available in this menu are discussed below (in order from the top of the menu to the bottom).

**
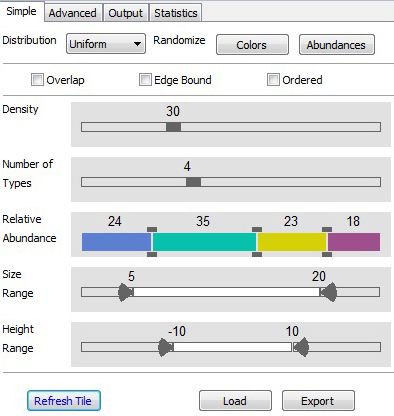
**

Distribution: Variables can be selected from two distributions: Gaussian or Uniform. When the uniform distribution is used the Size and Height Ranges signify the range of values from which random variables are uniformly chosen. When the Gaussian distribution is used the Size and Height Ranges signify the mean and standard deviation of the Gaussian distribution. This is evident in the Advanced menu, where the text within the menu changes to reflect mean and standard deviation, but in the Simple menu the sliders are used, whereby the leftmost slider adjusts the mean and the rightmost slider adjusts the standard deviation. (Additional information can be found on page 13).


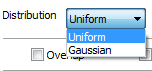

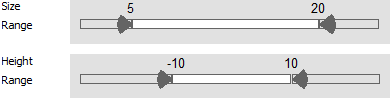


Randomize: The randomize part of the menu allows the user to re-randomize the colors and/or abundances of the current substrate. A click on the Colors button or will randomly change the colors in the relative abundance bar below. A click on the Abundances button will randomly change the relative abundances in the bar below. Changing the colors is cosmetic, while re-randomizing the abundances physically alters the tile. The Refresh button must be clicked for these changes to be transferred to a new tile.


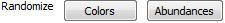


Overlap: When the Overlap checkbox is ticked substrates will have overlapping objects. When it is unticked there will be no overlaps.


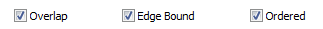


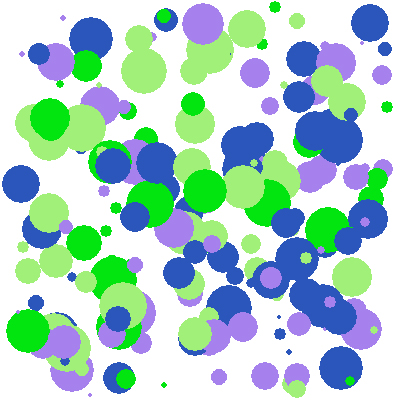


Edge Bound: When the Edge Bound button is ticked the objects will be constrained by the edge of the substrate. When it is unticked objects can be partially on the tile and partially off.


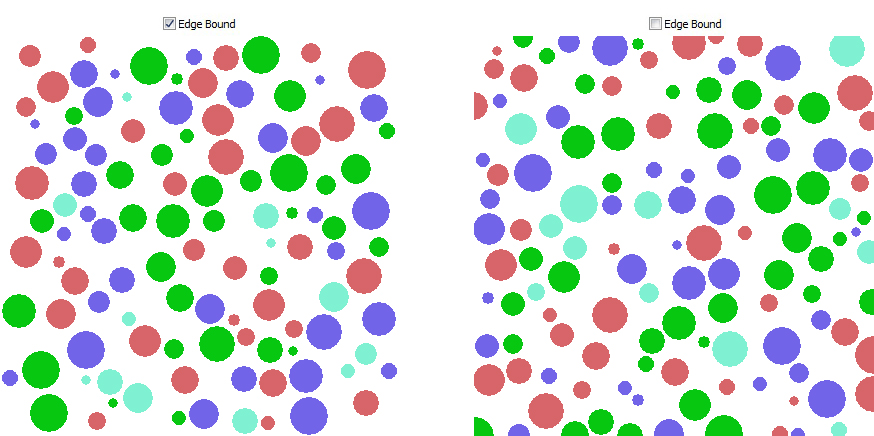


Ordered: When the Ordered checkbox is ticked the objects will appear in a grid pattern.


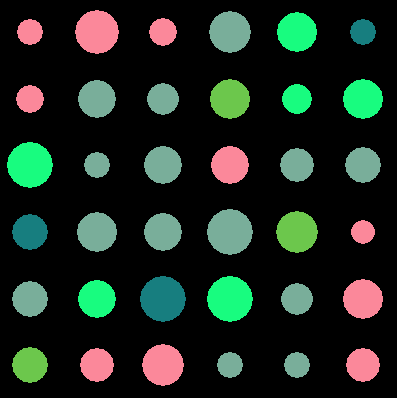


Density: The density slider enables the user to select the density of objects on the tile. *Note: the higher the density the longer the program will take to compute the substrate. If the substrate is not reproducible within the default time limit of 5 seconds, then the status ‘INCOMPLETE’ will be displayed (refer to page 14 for more information on the ‘Timeout’ function and page 19 for more details related to ‘Troubleshooting’).

Number of Types: The number of types slider enables the user to select the number of different types of objects on the substrate. The different types are signified by different colors.

Relative Abundance: The relative abundance slider enables the user to change the ratios of the different types of object on the substrate. Initially, these abundances are set randomly, but the user can change them manually as well as re-randomize them using the Randomize function explained on page 10.


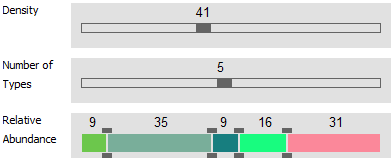


Size Range: Size range slider enables the user to select the size of the objects on the substrate. See the Distribution function on page 9 to read more about how sizes are selected from the given range.

Height Range: Height range slider enables the user to select the size of the objects on the substrate. See the Distribution function on page 9 to read more about how sizes are selected from the given range.


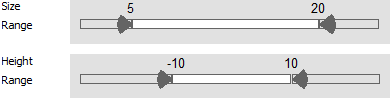


**7. Advanced Menu**

The Advanced Menu enables the user to set more complex parameters needed to make a substrate. These parameters will be discussed in order from the top of the menu to the bottom.

**
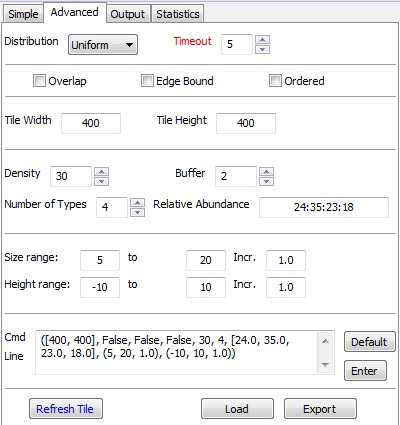
**

Distribution: Variables can be selected from two distributions: Gaussian or Uniform. When the uniform distribution is used the size and height entries specify Min, Max and Incr. (‘increment’). When the Gaussian distribution is used the size and height entries signify the Mean, StDev (‘standard deviation’) and Incr. (‘increment’) of the Gaussian distribution. For example if the uniform distribution is selected and the size minimum is 1, size maximum is 3 and increment is 1, then the sizes of the objects will either be 1, 2 or 3. If the Gaussian distribution is selected and the size mean is 4, standard deviation is 1 and increment is 1, then the sizes of the objects will be selected from a Gaussian distribution with those characteristics.


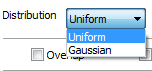

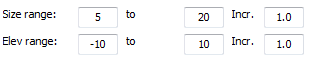
<--Uniform


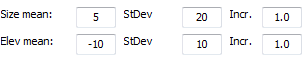
<--Gaussian

Timeout: Timeout is a special function which should generally not be altered. The Timeout is the amount of time in seconds allowed for computing a substrate. If the Timeout is exceeded before the program is able to finish placing objects on the tile, an ‘INCOMPLETE’ status will be shown in the lower right corner of the program. To avoid this, alter the substrate parameters or increase the Timeout. See the Troubleshooting section on page 19 for more information.


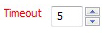


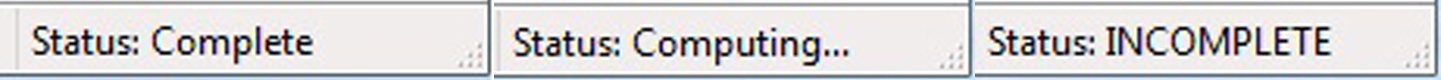


Overlap, Edge Bound, and Ordered buttons here function as they do in the Simple Menu (see page 10-11).


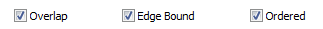


Tile Width & Tile Height: The default width and height is 400 x 400 generic units (i.e. it is scale free). The user can input any integer values for width and height and the substrate will fill the substrate viewer with the given rectangle; e.g. inputting 10 x 10 will produce a substrate that looks to be the same size as 400 x 400. This tool can be useful for producing actual substrates, for instance, if the substrate is eventually to be 200 mm x 200 mm then setting the width and height to 200 x 200 would result in all other measurements effectively being in millimeters.


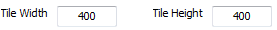


Density: The density slider enables the user to select the object density on the tile. *Note: the higher the density the longer the substrate will take to be completed.

Buffer: The buffer sets the minimum distance allowed between objects on the substrate. The bigger the buffer value the more spaced apart objects become.


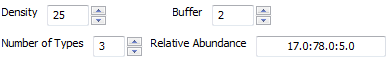


Number of Types, Relative Abundance, Size Range, Height Range here function as they do in the Simple Menu, except that exact values can be entered.


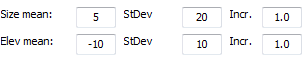


Cmd Line: The Command Line is used for debugging purposes and most users will not have a need for it. The command line allows the user to see the exact parameters being fed into the program. The command used to create each tile is saved when a substrate is saved. This enables users to recreate substrates using the original parameter settings.


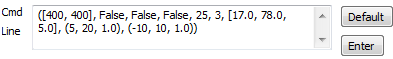


**8. Output Menu**

The Output Menu allows the user to view details about individual objects on the substrate. The details displayed are ID, Type, Position, Size and Height. Using the mouse to click on an object in the viewing pane will result in a highlighted line of corresponding details in a table in the Menu pane.

The object details will also appear above the list with entry fields which can be edited by the user. The selected object can be removed from the substrate (and the list) or it can be updated to reflect edited values entered by the user. In the case of overlapping objects, the user can step through the various selected objects using the arrow button on the output menu.

**
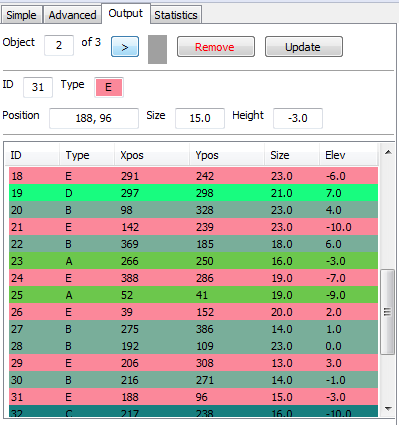

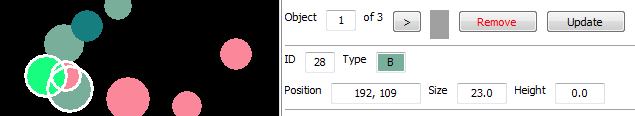
**

**
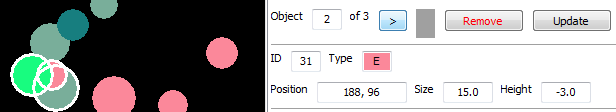
**

**9. Statistics Menu**

The Statistics Menu displays aggregate statistics on the various object types as well as overall substrate complexity.

**
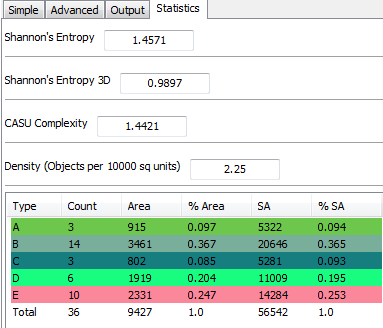
**

Shannon’s Entropy (*H*) is computed using the equation:


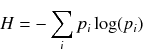


where *i* refers to each object type and *p* is the proportion each object type makes up of the total number of individuals.

“Shannon’s Entropy 3D (*H*-3D)” is computed with the same equation as Shannon’s Entropy, but it uses proportion of the surface area (SA) instead of proportion of individuals.

“CASU Complexity” is computed by multiplying Shannon’s Entropy by Shannon’s Entropy 3D.

**10. 3-Dimensional Modeling**

*CASU* gives users the option of viewing substrates in 3-D using the STL file format. This file is created when the user selects
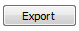
. Users can install a free STL viewing program (see below) and open it to view. In addition to viewing, it is also a compatible with stereolithography or 3D printing which can be used in rapid prototyping, manufacturing, and other applications to create physical models.


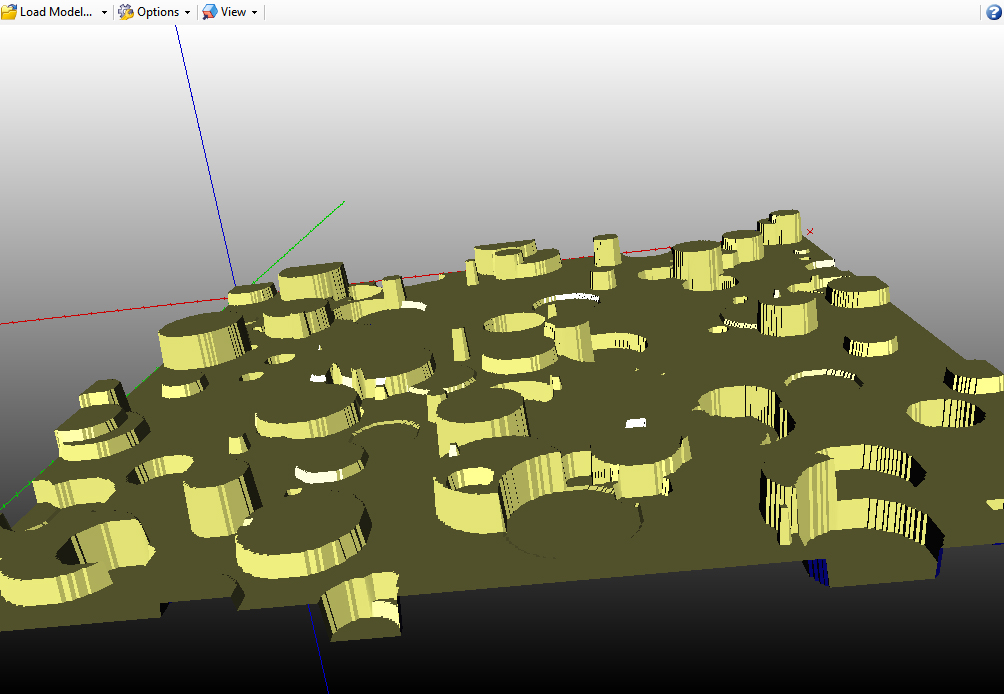


The following steps demonstrate how to view the substrate in 3-D:

[1] Download a free STL viewing program (e.g. STLView: <http://www.freestlview.com/>);

[2] Open the program (e.g. STLView);

[3] Click on the "Load Menu" option in the toolbar, then select "Add Model";

[4] Navigate to one of your STL files and click on that;

[5] You should be able to see it in the STL viewing program (it might take a few seconds to load).

**11. Troubleshooting**

Status: On occasion the program might be unresponsive while in the process of ‘Computing’ the substrate. Check the Status in the lower right corner of the program to see whether it has ‘Completed’ the computation. If the Status does not reach the ‘Completed’ state after the number of seconds specified in the Timeout parameter it will indicate that it is ‘INCOMPLETE’.


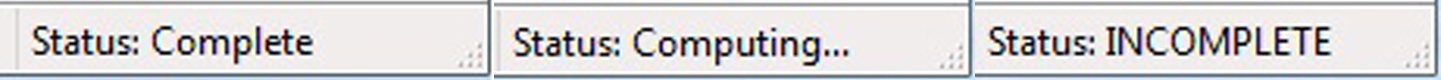


­­­This occurs when the Overlap parameter is not selected and the density is set too high. Due to the non-overlapping prerequsite, the program can require multiple iterations to place a given object. As the object density increases the likelihood of finding non-overlapping coordinates decreases, thereby increasing the time necessary to place each object. To avoid the error and complete the substrate the user can do one of two things:

1. Increase the time limit: to increase the time limit the user needs to alter the Timeout (refer to section on Timeout) parameter in the top section of the Advanced Menu. By default the Timeout is set to 5 seconds.
2. Alter the substrate parameters: allowing overlaps or decreasing the density and increasing the size range will increase the likelihood of the substrate completing within a reasonable time limit.

Statistics: By manually setting the ratios indicating the relative abundance of the object types to zero, users will get “nan” rather than a statistic. This will not happen unless users manually set the abundance of an object type to zero and cause the error.

Warning messages: Any settings altered in the Advanced tab will not be reflected in the Simple tab hence the pop-up message below. Although changes made in the Output tab is not displayed in the list directly below it, those changes will be reflected in the output files saved as well as the substrate viewer; so users can manipulate the individual objects to their desired specifications.

­
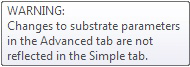


**12. Code Reference**

The source can be found in the file called CASU-source.zip. The code is written in Python.

The source code can also be downloaded at:

**13. Acknowledgements**

Many thanks to Toh Kok Ben for piloting the initial algorithms that helped shape *CASU* and to Alex Wong for adopting our ideas, providing guidance and generously coding the first few versions of the program. This study was supported by the Singapore Delft Water Alliance (SDWA) JBE-B's "Towards designing innovative coastal protection using ecosystem-based approaches; deriving underlying ecological knowledge" (grant number R-303-001-021-414).

**14. References**

Bell S. S., Fonseca M. S. & Motten L. B., 1997. Linking ecological restoration and landscape ecology. *Restoration Ecology* **5**: 318–323.

Cairns, J., Jr., ed. 1982. *Artificial Substrates*. Ann Arbor Science Publishers, Inc., Ann Arbor, MI. 278 pp.

Loke, L. H. L., Clews, E. Low, E., Belle, C. C.,Todd,P. A., Eikaas, H. A., & Ng, P. K. L., 2010. Methods for sampling benthic macroinvertebrates in tropical lentic systems. *Aquatic Biology* **10**:119-130

Matias, M. G., Underwood, A. J., Hochuli, D. F., & Coleman, R. A., 2010. Independent effects of patch size and structural complexity on diversity of benthic macroinvertebrates. *Ecology* **91**(7): 1908-1915.

Palmer, M. A., Menninger, H. L. & Bernhardt, E. 2009. River restoration, habitat heterogeneity and biodiversity: a failure of theory or practice? *Freshwater Biology* **55**: 1-18.

Pianka, E. R., 2000. *Evolutionary ecology*, 6^th^ edition. Benjamin Cummings, San Francisco. 512pp.

Pratt, J. R. 1994. Artificial habitats and ecosystem restoration: managing for the future. *Bulletin of Marine Science* **55**: 268-275.

Ritchie, M. E. & H. Olff, 1999. Spatial scaling laws yield a synthetic theory of biodiversity. *Nature* ***400***: 557-560.
